# WISP1 mediates IL-6-dependent proliferation in primary human lung fibroblasts

**DOI:** 10.1038/srep20547

**Published:** 2016-02-12

**Authors:** S. Klee, M. Lehmann, D. E. Wagner, H. A. Baarsma, M. Königshoff

**Affiliations:** 1 Comprehensive Pneumology Center, Helmholtz Zentrum München, Munich, Germany.; 2 Member of the Ludwig-Maximilians-Universität, University Hospital Grosshadern and the German Center of Lung Research (DZL), Nußbaumstraße 20, 80336 Munich, Germany.

## Abstract

Idiopathic pulmonary fibrosis (IPF) is a progressive and fatal interstitial lung disease. IPF is characterized by epithelial cell injury and reprogramming, increases in (myo)fibroblasts, and altered deposition of extracellular matrix. The Wnt1-inducible signaling protein 1 (WISP1) is involved in impaired epithelial-mesenchymal crosstalk in pulmonary fibrosis. Here, we aimed to further investigate WISP1 regulation and function in primary human lung fibroblasts (phLFs). We demonstrate that WISP1 is directly upregulated by Transforming growth factor β1 (TGFβ1) and Tumor necrosis factor α (TNFα) in phLFs, using a luciferase-based reporter system. WISP1 mRNA and protein secretion increased in a time- and concentration-dependent manner by TGFβ1 and TNFα in phLFs, as analysed by qPCR and ELISA, respectively. Notably, WISP1 is required for TGFβ1- and TNFα-dependent induction of interleukin 6 (IL-6), a mechanism that is conserved in IPF phLFs. The siRNA-mediated WISP1 knockdown led to a significant IL-6 reduction after TGFβ1 or TNFα stimulation. Furthermore, siRNA-mediated downregulation or antibody-mediated neutralization of WISP1 reduced phLFs proliferation, a process that was in part rescued by IL-6. Taken together, these results strongly indicate that WISP1-induced IL-6 expression contributes to the pro-proliferative effect on fibroblasts, which is likely orchestrated by a variety of profibrotic mediators, including Wnts, TGFβ1 and TNFα.

Idiopathic pulmonary fibrosis (IPF) is a devastating and progressive interstitial lung disease with a median survival of 3 to 5 years and limited therapeutic options^1^,^2^. Recently, two drugs (Pirfenidone and Nintedanib) have been approved for the treatment of mild/moderate IPF, both of which significantly reduce lung function decline in IPF patients^3^,^4^. Therapies halting or reversing the disease progression are lacking and thus a more in-depth understanding of pathomechanisms driving IPF is needed. Histopathological features of IPF include alveolar epithelial cell injury and hyperplasia, (myo)fibroblast proliferation and differentiation, along with increased extracellular matrix (ECM) production and deposition^2^,^5^,^6^. Fibroblast foci are a key histologic characteristic of IPF and a major site of fibroblast proliferation^7^. As such, IPF is likely driven by impaired epithelial and mesenchymal cell communication. The Wnt1-inducible signaling protein 1 (WISP1) is a member of the CCN (**C**yR61, **C**TGF, **N**OV) family of matricellular proteins, which have been reported to be critically involved in epithelial-mesenchymal crosstalk^8^,^9^. WISP1 has been implicated in lung and airway remodeling^10^,^11^,^12^. Moreover, WISP1 is highly upregulated in patients with IPF as well as in experimental lung fibrosis^13^,^14^,^15^. Importantly, neutralizing antibodies against WISP1 attenuated the development of bleomycin-induced pulmonary fibrosis *in vivo*, thus demonstrating the potential of WISP1 as a therapeutic target for IPF^13^.

Other cytokines involved in disturbed cellular crosstalk in IPF are Transforming growth factor β1 (TGFβ1) and Tumor necrosis factor α (TNFα). Both cytokines are highly upregulated in IPF and alter ECM production, survival and proliferation of distinct cell types in the lung, including alveolar epithelial cells and lung fibroblasts^2^,^16^. Consistently, knockout mice for TGFβ1, TNFα or components of their respective downstream signaling pathways attenuated development of experimental lung fibrosis^16^,^17^.

We have recently reported that IPF fibroblasts display increased WISP1 levels and that miRNAs regulate WISP1 expression in TGFβ1-primed fibroblasts^15^. Here we aimed to further elucidate the upstream regulation of WISP1 in a profibrotic environment as well as its downstream functions in primary human lung fibroblasts. We demonstrate that WISP1 is directly upregulated by both TGFβ1 and TNFα in primary human lung fibroblasts and that the presence of WISP1 is required for TGFβ1- and TNFα-induced interleukin 6 (IL-6) production. Moreover, we show that WISP1-induced IL-6 contributes to increased fibroblast proliferation.

## Results

### WISP1 is a common downstream target of profibrotic signaling mediators

**Figure 1:**
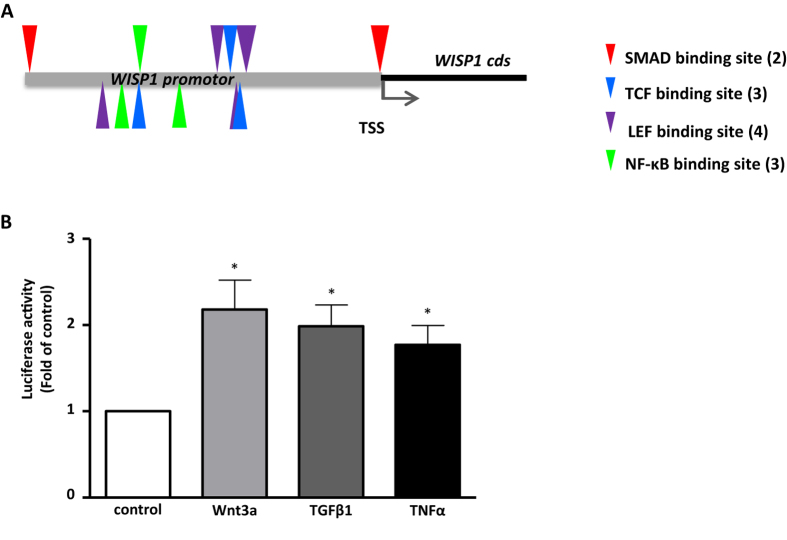
The WISP1 promoter region contains potential binding sites for transcription factors activated by profibrotic cytokines. (**A**) *In silico* analysis of the WISP1 promoter (2.5 kb upstream of the WISP1 transcription start site (TSS)) revealed potential binding sites for SMAD, TCF, LEF and NF-κB. (**B**) A reporter construct containing the WISP1 2.5 kb promoter region was transfected into primary human lung fibroblasts (phLFs). phLFs were treated with 100 ng/ml Wnt3a, 2 ng/ml TGFβ1 or 10 ng/ml TNFα for 24 hours followed by measurement of luciferase activity. Wnt3a, TGFβ1 and TNFα all significantly induced luciferase activity as compared to unstimulated conditions (n = 4, *p < 0.05, 1-way ANOVA followed by Neuman-Keuls multiple comparison test).

The matricellular protein WISP1 has recently been reported to be increased in IPF-derived lung fibroblasts^15^. Here, we addressed the question whether WISP1 expression is regulated by specific profibrotic mediators in primary human lung fibroblasts (phLFs). *In silico* analysis of a region of a total of 2.5 kb upstream of the WISP1 transcription starting site (here called WISP1 promoter region) revealed potential binding sites for transcription factors like T-cell factor (TCF) and lymphoid enhancer factor (LEF), SMADs, as well as nuclear factor kappa B (NF-κB), which are activated by canonical Wnt, TGFβ1 and TNFα signaling, respectively (Fig. 1A). In order to verify these potential mediators, we transfected phLFs with either a luciferase-based reporter plasmid containing the 2.5 kb WISP1 promoter element or a control plasmid and subsequently treated the phLFs with TGFβ1, TNFα, or Wnt3a. Wnt3a has been reported to exhibit profibrotic effects in the lung^18^ and was further used as a positive control, since WISP1 has been described to be β-catenin dependent^13^,^19^. As shown in Fig. 1B, treatment with all three profibrotic cytokines induced a significant increase in luciferase activity, indicating that in addition to Wnt3a, TGFβ1 and TNFα directly induce WISP1 in phLFs.

### TGFβ1 induces WISP1 expression and secretion in primary human lung fibroblasts

**Figure 2:**
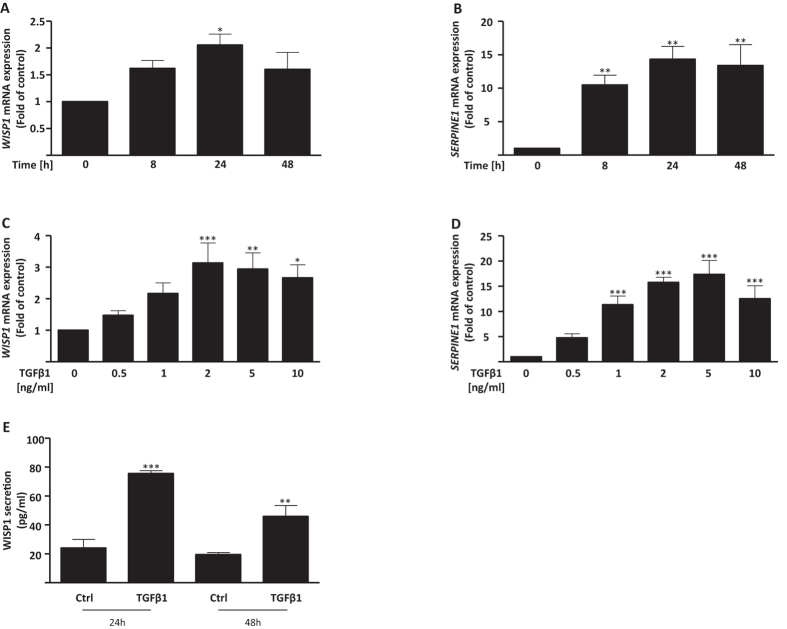
TGFβ1 induces WISP1 in primary human lung fibroblasts (phLFs). phLFs were treated with TGFβ1 (2 ng/ml) for 8, 24 and 48 hours and the expression of (**A**) *WISP1* and (**B**) *SERPINE1* were analysed using RT-qPCR. The phLFs were treated with TGFβ1 concentrations ranging from 0.5 to 10 ng/ml and the expression of (**C**) *WISP1* and (**D**) *SERPINE1* were analysed using RT-qPCR at 24 hours. *WISP1* was significantly upregulated using 2 ng/ml at 24 hours while *SERPINE1* was upregulated at 8 hours and at all further timepoints. (**E**) phLFs were treated with TGFβ1 (2 ng/ml) for 24 and 48 hours and WISP1 secretion was significantly upregulated as measured by ELISA (n = 3–6; *p < 0.05; **p < 0.01; ***p < 0.001; 1-way ANOVA followed by Neuman-Keuls multiple comparison test; compared to respective control).

TGFβ1 is the main profibrotic cytokine active in IPF. It is involved in numerous processes including proliferation and ECM production by fibroblasts as well as epithelial cell reprogramming, which altogether ultimately drive lung fibrosis progression. We have recently shown that miRNAs regulate WISP1 expression in a TGFβ1-driven environment^15^. Here, we investigated the dynamics of WISP1 regulation by TGFβ1 in more detail. The induction of *WISP1* expression by TGFβ1 was time- and concentration-dependent (Fig. 2A,C; 24 hours: 1.92 ± 0.23 fold over control; 2 ng/ml TGFβ1: 3.14 ± 0.64 fold over control). Notably, the induction of *WISP1* was similar to the induction of *SERPINE1* (Fig. 2B,D), a direct target gene of TGFβ1. Next, we investigated the effect of TGFβ1 on WISP1 protein levels, and found significantly increased WISP1 secretion in phLFs as early as 24 hours upon TGFβ1 stimulation (Fig. 2E; 24 hours: control vs. 2 ng/ml TGFβ1: 24.02 ± 5.88 pg/ml vs. 75.52 ± 1.98 pg/ml; 48 hours: control vs. 2 ng/ml TGFβ1: 19.52 ± 1.38 pg/ml vs. 45.87 ± 7.63 pg/ml). Thus, TGFβ1 induces *WISP1* mRNA expression and secretion in a time- and concentration-dependent manner in phLFs.

### WISP1 is regulated by TNFα in primary human lung fibroblasts

**Figure 3:**
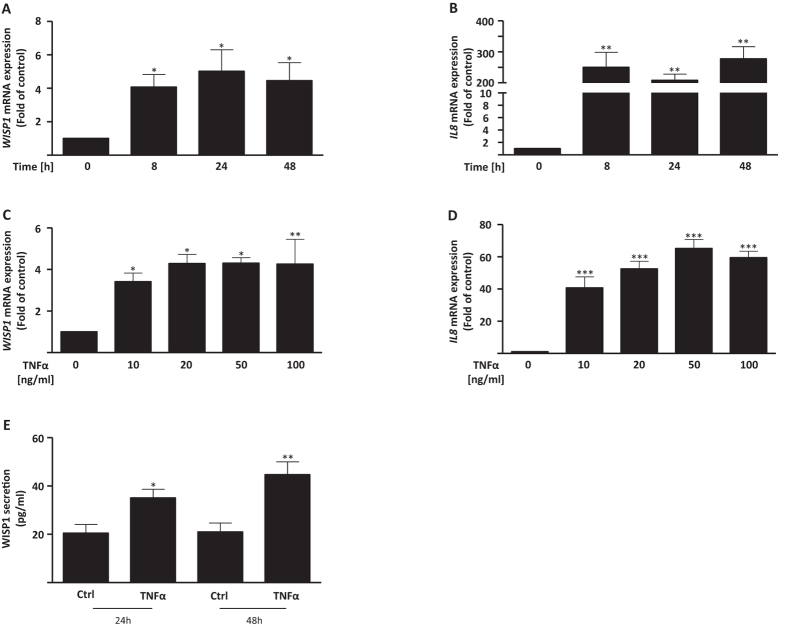
TNFα induces WISP1 in primary human lung fibroblasts (phLFs). The treatment of phLFs with TNFα (10 ng/ml) for 8, 24 and 48 hours was followed by the analysis of the expression of (**A**) *WISP1* and (**B**) *IL8* using RT-qPCR. (**C,D**) phLFs were treated with TNFα concentrations from 10 to 100 ng/ml and the expression of *WISP1* and *IL8* was analysed using RT-qPCR. *WISP1* and *IL8* were significantly increased after 8 hours of TNFα stimulation with 10 ng/ml (**E**) WISP1 secretion by phLFs after treatment with TNFα (10 ng/ml) for 24 and 48 hours was significantly increased as measured by ELISA (n = 3–4; *p < 0.05; **p < 0.01; ***p < 0.001; 1-way ANOVA followed by Neuman-Keuls multiple comparison test; compared to respective control).

TNFα is a multi-faceted cytokine with numerous functions and is associated with lung fibrosis^20^,^21^. Our promoter studies suggested that WISP1 is a direct target of TNFα (Fig. 1). Next, we investigated the dynamics of WISP1 regulation by TNFα in more detail. We found that TNFα induced *WISP1* mRNA expression as early as 8 hours upon stimulation with a near-maximal induction of *WISP1* at 10 ng/ml (Fig. 3A,C; 8 hours: 4.61 ± 0.68 fold over control; 10 ng/ml: 3.41 ± 0.43 fold over control) accompanied by a similar trend of induction of the known TNFα target gene *IL-8* (Fig. 3B,D). Moreover, enhanced WISP1 protein secretion was observed at 24 and 48 hours upon TNFα stimulation (Fig. 3E; control vs. 10 ng/ml TNFα at 24 hours: 20.47 ± 3.66 pg/ml vs. 35.02 ± 3.63 pg/ml; control vs. 10 ng/ml TNFα at 48 hours: 20.99 ± 3.81 pg/ml vs. 44.7 ± 5.4 pg/ml).

We next sought to explore common downstream mechanisms by which TGFβ1 and TNFα might exert their effects to upregulate *WISP1* expression. We found that TGFβ1- and TNFα-mediated induction of *WISP1* was primarily NF-κB-dependent as shown by a significant reduction of *WISP1* in the presence of the IKKβ inhibitor (SC-514; Suppl. Fig. 1), but independent of extracellular signal-related kinase (MEK1/2; inhibitor: U0126) or c-Jun N-terminal kinase 1/2 (JNK1/2; inhibitor: SP600125), respectively.

### WISP1 is required for IL-6 expression by the profibrotic cytokines TGFβ1 and TNFα

**Figure 4:**
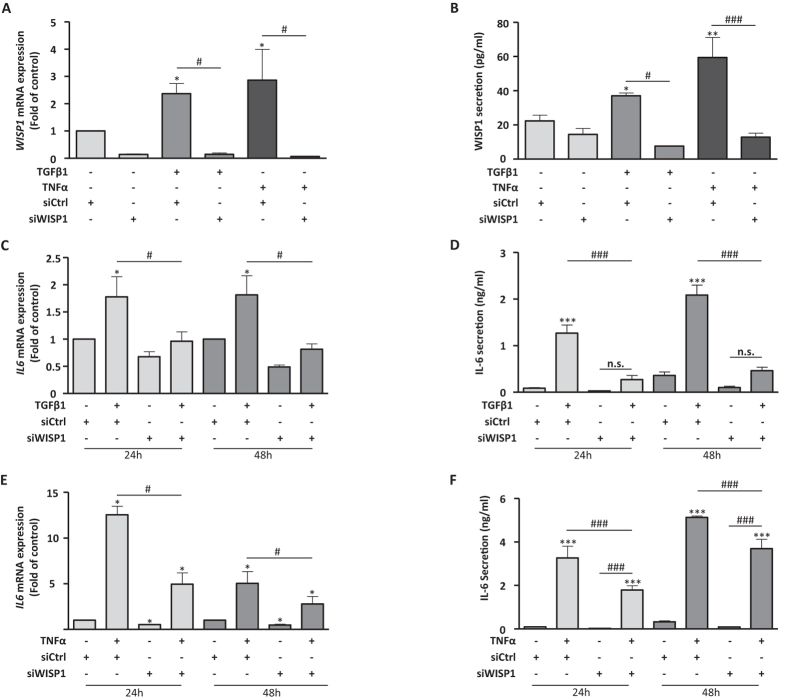
Loss of WISP1 results in reduced expression of TGFβ1- and TNFα-induced IL-6 in phLFs. phLFs were transfected with control (siCtrl) or WISP1-targeting (siWISP1) siRNAs and subsequently treated with TGFβ1 (2 ng/ml) or TNFα (10 ng/ml) for (**A,B**) 24 hours or (**C-F**) 24 and 48 hours. *WISP1* (**A**) mRNA was analysed by RT-qPCR and (**B**) secretion was measured by ELISA. siRNA treatment against *WISP1* results in significant loss of WISP1 at baseline and in the presence of TGFβ1 and TNFα treatment. IL-6 levels were analysed at 24 and 48 hours of 2 ng/ml TGFβ1 or 10 ng/ml TNFα treatment and (**C,E**) IL-6 mRNA was analysed by RT-qPCR and (**D,F**) IL-6 secretion was measured by ELISA. Loss of WISP1 results in loss of IL-6 induction even in the presence of TGFβ1 or TNFα treatment (n = 4; *^,#^p < 0.05; **p < 0.01; ***^,###^p < 0.001; 1-way ANOVA followed by Neuman-Keuls multiple comparison test).

Since both TGFβ1 and TNFα induced WISP1 in phLFs, we next asked the question if WISP1 in turn is involved in TGFβ1- or TNFα-dependent cellular signaling and function. We analysed interleukin 6 (IL-6), which is induced by both TGFβ1 and TNFα in various cell types, e.g. via NF-κB^22^,^23^,^24^. IL-6 is a pro-inflammatory and pro-fibrotic cytokine reported to be involved in the pathogenesis of IPF^25^,^26^. To address the question if WISP1 is involved in *IL-6* induction in primary human lung fibroblasts, we used an siRNA-based approach to downregulate WISP1 prior to stimulation with TGFβ1 (2 ng/ml) or TNFα (10 ng/ml), respectively. *WISP1* was effectively downregulated upon specific siRNA knockdown by 86.2 ± 2.4% after 24 h in the unstimulated condition and importantly, also in the presence of TGFβ1- or TNFα-stimulation (Fig. 4A, baseline: −86.2 ± 2.4%, TGFβ1: −85.6 ± 3.7%, TNFα: −93.6 ± 0.5%; compared to siCtrl). We further validated the knockdown on protein level by analysing WISP1 secretion and found a strong reduction in WISP1 secretion upon siRNA-mediated knockdown in the presence of either TGFβ1 or TNFα (Fig. 4B; siCtrl + TGFβ1 vs. siWISP1 + TGFβ1: 37.02 ± 1.75 pg/ml vs. 7.62 ± 0.45 pg/ml; siCtrl + TNFα vs. siWISP1 + TNFα: 59.46 ± 10.82 pg/ml vs. 12.82 ± 2.45 pg/ml). We next examined the induction of IL-6 production by phLFs upon TGFβ1 or TNFα stimulation in the presence and absence of WISP1. Both TGFβ1 and TNFα treatments led to a significant upregulation of *IL6* expression in phLFs after 24 and 48 hours of stimulation (Fig. 4C,E; TGFβ1 at 48 hours: 1.81 ± 0.36 fold over control; TNFα at 48 hours: 5.04 ± 1.29 fold over control). In the absence of WISP1, however, IL-6 expression and secretion was significantly lower compared to the TGFβ1- or TNFα-treated cells transfected with control siRNA (Fig. 4C,E, respectively; siCtrl + TGFβ1 vs. siWISP1 + TGFβ1: *IL6* mRNA 1.81 ± 0.36 fold over control vs. 0.81 ± 0.1 fold over control; siCtrl + TNFα vs. siWISP1 + TNFα: *IL6* mRNA 5.04 ± 1.29 fold over control vs. 2.78 ± 0.81 fold over control). Importantly, these results were validated on the protein level by analysing IL-6 secretion (Fig. 4D,F, respectively; siCtrl + TGFβ1 vs. siWISP1 + TGFβ1 at 48 h: 2.09 ± 0.21 ng/ml vs. 0.46 ± 0.09 ng/ml; siCtrl + TNFα vs. siWISP1 + TNFα at 48 h: 5.13 ± 0.06 ng/ml vs. 3.69 ± 0.44 ng/ml). Importantly, we found similar results in phLFs derived from IPF patients, suggesting that the same mechanisms of WISP1 induction as well as WISP1-dependent *IL6* expression are present in IPF-derived phLFs (Suppl. Fig. 2). Notably, WISP1 specifically affected IL-6 production in phLFs, while the induction of IL8 by TNFα, as well as other cytokines, such as monocyte chemoattractant protein-1 (MCP-1) and interferon γ (IFNγ) were not affected by the loss of WISP1 as measured by a multiplex ELISA (Suppl. Fig. 3A,B and Suppl. Tables 1 and 2).

### Loss of WISP1 reduces proliferation of primary human lung fibroblasts

**Figure 5:**
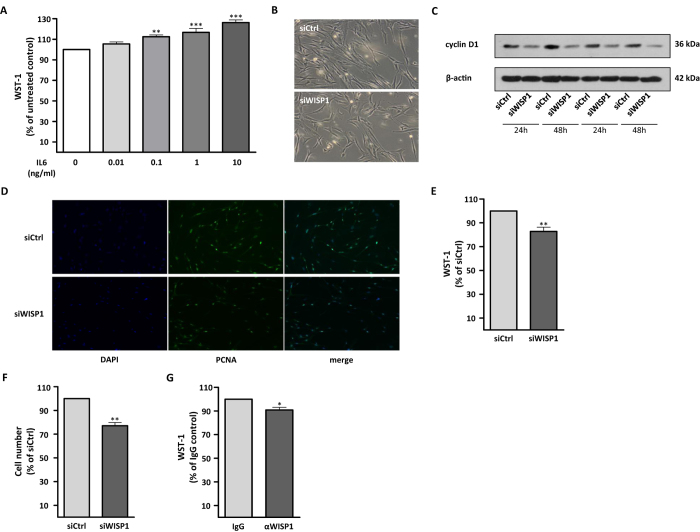
Loss of WISP1 decreases proliferation of phLFs. (**A**) The phLFs showed a dose-dependent increase in proliferation with different concentrations of IL-6 as measured by WST-1. (**B–F**) phLFs transfected with control (siCtrl) or WISP1-targeting (siWISP1) siRNA had decreased proliferation. (**B**) Representative bright field images of siCtrl and siWISP1 transfected cells (magnification: 100×). (**C**) Representative Western Blot of cyclin D1 levels in phLFs after 24 and 48 hours of treatment with siWISP1 and (**D**) immunofluorescence staining of PCNA (green) and DAPI (blue; magnification: 100×) shows qualitative decreases in cell number and PCNA staining. Decreased proliferation in siWISP1 conditions was measured by (**E**) WST-1 assay and (**F**) cell count. (**G**) Additionally, phLFs were treated with a neutralizing αWISP1 antibody and decreased proliferation was observed by WST-1 assay (n = 3–7; *p < 0.05; **p < 0.01; A: 1-way ANOVA followed by Neuman-Keuls multiple comparison test; **E**–**G**: Student’s T-test).

IL-6 has been reported to exhibit pro-proliferative effects on lung fibroblasts^27^,^28^. In support of these findings, stimulating phLFs with IL-6 led to a concentration-dependent increase in cell proliferation (Fig. 5A), however, this effect was not mediated by an increase in WISP1 following IL-6 treatment since IL-6 did not induce *WISP1* expression in phLFs (Suppl. Fig. 4A). As WISP1-depleted cells produced less IL-6 and WISP1 has been shown to be pro-proliferative in non-lung cells^8^ as well as in lung alveolar epithelial type II (ATII) cells *in vitro*^13^, we investigated the effect of WISP1 on the proliferation of phLFs. Indeed, siRNA-mediated knockdown of WISP1 resulted in a significantly reduced number of phLFs and reduced expression of cyclin D1 as analysed by Western Blotting (Fig. 5B,C). Moreover, we found decreased immunofluorescent staining of Proliferating-Cell-Nuclear-Antigen (PCNA; Fig. 5D). In addition, we observed significantly reduced metabolic activity of phLFs in a WST-1 assay due to loss of WISP1 (Fig. 5E, reduction by 18.7 ± 3.5%, Suppl. Fig. 4B,C) and reduced cell numbers compared to control siRNA transfected cells (Fig. 5F; reduction by 22.9 ± 2.8%). Moreover, using either a neutralizing antibody targeting WISP1 (Fig. 5G) or an IKKβ inhibitor (SC-514) that downregulates *IL6* expression (Suppl. Fig. 5A,B), we further corroborated our results and observed a significantly decreased metabolic activity of phLFs upon αWISP1 treatment (Fig. 5G; reduction by 9.2 ± 2.4%) as well as upon NF-κB inhibition (Suppl. Fig. 5C; reduction by 23.5 ± 2.4%). Taken together, our data strongly indicate that WISP1 exhibits pro-proliferative effects on phLFs. To this end, we further found that *WISP1* expression levels in IPF tissue negatively correlated with lung function measurement parameters (%DL_CO_ and %FVC), which also have been shown to correlate to the number of fibroblast foci in IPF^29^ (Suppl. Fig. 6A,B).

### WISP1-induced IL-6 expression contributes to primary human lung fibroblast proliferation

**Figure 6:**
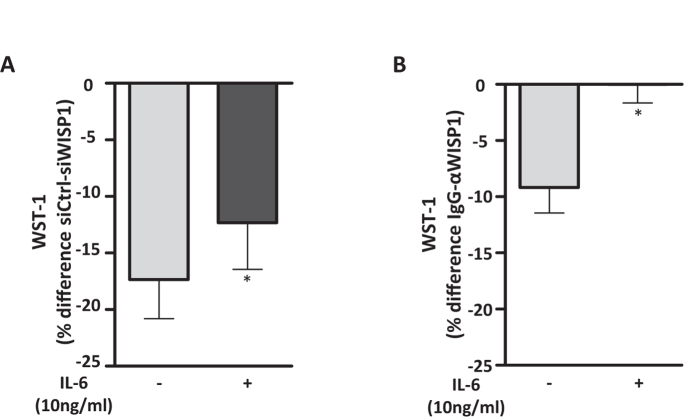
WISP1 increases proliferation of phLFs in part via IL-6. The phLFs were either (**A**) transfected with siCtrl or siWISP1 or (**B**) pre-incubated with a neutralizing αWISP1 antibody and treated with or without IL-6 (10 ng/ml). Metabolic activity of the phLFs was measured by WST-1 conversion and statistically significant increases were observed following IL-6 treatment demonstrating that IL-6 induction is, in part, responsible for mediating the pro-proliferative effects of WISP1 (n = 7; *p < 0.05; Student’s T-test).

**Figure 7:**
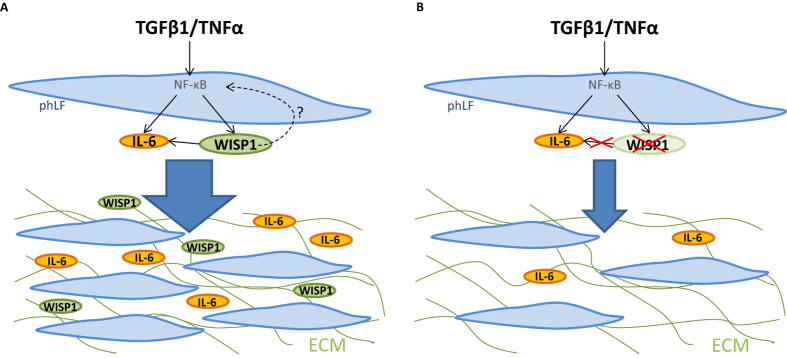
Proposed schematic model. (**A**) The profibrotic cytokines TGFβ1 and TNFα can induce WISP1 presumably via NF-κB in phLFs, which results in WISP1-dependent IL-6 production and increased proliferation of phLFs. (**B**) In the absence of WISP1, decreased IL-6 levels lead to reduced fibroblast proliferation. Our working hypothesis is that WISP1 controls IL-6 expression via a positive feedback on NF-κB.

Given our findings that the presence of WISP1 is required for the induction of IL-6 by TGFβ1 and TNFα and that IL-6 is able to induce proliferation in phLFs, we next hypothesized that the reduced proliferation of phLFs in the absence of WISP1 might be a result of reduced IL-6 levels. To address this question, we either transfected phLFs with siRNAs (siWISP1 and respective control) or treated cells with a neutralizing αWISP1 antibody and subsequently treated the cells with IL-6 (10 ng/ml; Fig. 6A,B). Notably, cells lacking WISP1 showed a significantly higher increase in the proliferative response to IL-6 compared to siCtrl transfected cells (Fig. 6A; 16.8% vs. 6.3%). Consistently, the decrease in WST1 by WISP1 siRNA was in part restored by IL-6 (Fig. 6A; untreated vs. IL-6 treated: −17.34 ± 3.5% vs. −12.32 ± 4.12%), indicating a partial rescue of the proliferation defect by IL-6 in cells lacking WISP1. Additionally, cells treated with the αWISP1 antibody and subsequently with IL-6 showed fully restored proliferation capacity compared to cells treated with an IgG control (Fig. 6B; untreated vs. IL-6 treated: −9.2 ± 2.4% vs. 0 ± 1.8%). Taken together, these results strongly indicate that WISP1-induced IL-6 expression significantly contributes to the pro-proliferative fibroblast function, which is likely orchestrated by a variety of profibrotic mediators, including Wnts, TGFβ1 and TNFα (Fig. 7).

## Discussion

IPF is a chronic lung disease with poor outcome prediction^1^,^2^. Two recently approved drugs have been shown to reduce the progression of lung function decline in mild to moderate IPF, however, they have not been reported to halt or reverse pathological changes in lung architecture and lung function^3^,^4^. Thus, further understanding of the pathomechanisms involved in IPF development and progression is required to develop more effective therapeutic strategies. TNFα and TGFβ1 are highly upregulated in IPF and targeting profibrotic mediators induced downstream of TGFβ1 and TNFα represents a promising therapeutic approach for IPF^2^,^13^. Here, we show that both TGFβ1 and TNFα induce WISP1 expression and secretion in primary human lung fibroblasts. The WISP1 promoter contains transcription factor binding sites for TCF/LEF, SMADs, as well as NF-κB and our initial promoter studies indicate a direct control of *WISP1* expression by Wnt3a, TGFβ1, and TNFα. WISP1 is upregulated in IPF and has been shown to be an epithelial cell-derived mediator of impaired epithelial-to-mesenchymal crosstalk. Of note, neutralizing WISP1 led to a reduction of experimentally induced lung fibrosis^13^. WISP1 has further been described as a target gene of canonical Wnt signaling^30^, a developmental pathway reactivated in IPF^31^ and inhibition of which has been shown to prevent and reverse fibrotic changes in the murine lung^32^,^33^. We have recently reported that WISP1 is increased in IPF fibroblasts^15^. Here, we revealed a novel mechanism by which WISP1 contributes to profibrotic cellular fibroblast function and thus underline the potential of WISP1 as a therapeutic target for IPF.

IPF is characterized by increased fibroblast proliferation and accumulation along with ECM production. Thus, altering fibroblast function is of main interest as a potential therapeutic strategy in IPF. Pulmonary WISP1 has been shown to be highly expressed by alveolar epithelial type II (ATII) cells. Immunohistochemical analysis of WISP1 in IPF tissue specimen revealed only a weak staining in lung fibroblasts/interstitium of the lung^13^. Our *in vitro* data using primary human lung fibroblasts indicate that fibroblasts are an additional source for WISP1. While comparative analysis of human alveolar epithelial cells and phLFs revealed that the amount of WISP1 secreted by ATII cells *in vitro* exceeds the secretion by fibroblasts by about 25 fold (data not shown), it is likely that fibroblast-derived WISP1 might directly act on surrounding cells in the microenvironment of a fibroblast focus.

IL-6 is a well-described inducer of lung fibroblast proliferation^28^. Here, we found that WISP1 is required for TGFβ1- and TNFα-dependent induction of IL-6 in lung fibroblasts. We have recently described a link between Wnt/β-catenin signaling and interleukin secretion in pulmonary fibrosis^18^. Induction of Wnt/β-catenin signaling by Wnt3a in alveolar epithelial cells *in vitro* and *in vivo* resulted in a significant increase in IL-6. Moreover, IL-6 was shown to be upregulated in the bronchial alveolar lavage fluid (BALF) of IPF patients and in experimental lung fibrosis^18^,^25^. In this context, it has also been shown that a mutation of the IL-6 receptor subunit gp130, by which signal transduction downstream of gp130 is solely directed through signal transducer and activator of transcription 3 (STAT3) but no longer via ERK/MAPK signaling, led to a worsened fibrotic lung phenotype in mice upon bleomycin challenge. Interestingly, loss of IL-6 in gp130 mutated mice resulted in protection against bleomycin-induced lung fibrosis, indicating that IL-6 itself is necessary for fibrosis development in this model^34^. Altogether these data support the notion that WISP1 and IL-6 are inter-connected critical mediators contributing to IPF pathogenesis.

We further analysed functional effects of WISP1 and found that depletion of WISP1 by two independent approaches resulted in decreased fibroblast proliferation, thus further corroborating a pro-proliferative role of WISP1^35^,^36^,^37^,^38^. In support of these findings, we demonstrate that treatment of WISP1-deficient or WISP1-neutralized phLFs with IL-6, in part, rescued the effect on fibroblast proliferation. These data suggest that WISP1 increases proliferation of human lung fibroblasts via IL-6 induction (Fig. 7). It is also important to note that in our experimental setup IL-6 was not able to fully rescue fibroblast proliferation upon loss of WISP1 by siRNA mediated knockdown *in vitro*. Thus, it is likely that WISP1 potentially affects additional pro-proliferative factors. Some of these, such as IL-8, were included in our multiplex ELISA and were not affected by the loss of WISP1. Other cytokines belonging to the IL-6 interleukin subfamily, which includes IL-11, IL-31, Oncostatin M and Leukemia inhibitory factor (LIF) have been described as pro-proliferative^39^, however, they were not tested in our multiplex ELISA approach. Though there are no reports yet, linking WISP1 to any of these latter cytokines, one of these cytokines or a combination thereof might be responsible for the additional effect of WISP1 knockdown on proliferation. Future studies are needed to address these open questions.

We found that TGFβ1 and TNFα mainly induced WISP1 expression in phLFs via NF-κB pathway. Notably, the presence of WISP1 is required for *IL6* expression, which is also an NF-κB-driven target gene and induced by both TGFβ1 and TNFα. As such, we hypothesize that WISP1 has a positive feedback function on NF-κB-driven genes. WISP1 is a member of the CCN family, which also includes Cysteine-rich angiogenic inducer 61 (Cyr61), Connective tissue growth factor (CTGF) and Nephroblastoma overexpressed protein (NOV). These family members have been shown to induce downstream effects via NF-κB in different cell types^40^,^41^,^42^. In line with these findings, WISP1 was previously described to activate NF-κB in synovial fibroblasts^43^. These data strongly support our findings and suggest that WISP1 has a potential positive feedback function on IL-6 through the activation of NF-κB in primary human lung fibroblasts.

Currently, it is unknown, which cell-surface molecules are required for WISP1-mediated signaling in phLFs. However, CCN family members are known to signal through integrins^44^. Integrins are versatile cell surface receptors that, through various combinations of their alpha and beta subunits, can regulate a variety of different responses in a cell-specific manner^45^. Importantly, integrins play a role in the pathogenesis of IPF, both by functioning as receptors and by activating molecules like TGFβ1^45^. For example, integrin α_v_β_6_ has been shown to be important for TGFβ1 activation on lung epithelial cells, and β_6_ knockout animals or α_v_β_6_ neutralization attenuated pulmonary fibrosis development^45^. WISP1 has been reported to signal through α_v_β_5_ in synovial fibroblasts^43^, an integrin heterodimer that is also expressed on lung fibroblasts^46^ and thus represents a potential integrin involved in WISP1-induced IL-6 production in lung fibroblasts.

Taken together, our data show that WISP1 is a common downstream target of major pro-fibrotic factors, TGFβ1 and TNFα, in primary human lung fibroblasts. Moreover, WISP1 exerts its profibrotic functions through IL-6-dependent induction of fibroblast proliferation. These data further underline the importance of WISP1 in the progression of lung fibrosis and strengthen the potential benefit of an anti-WISP1 therapy in IPF patients.

## Materials and Methods

### Reagents

Recombinant TGFβ1 (human; 240-B/CF), recombinant TNFα (human, 210-TA/CF) and recombinant IL-6 (206-IL/CF) were purchased at R&D systems (Abingdon, UK).

### Cell culture

Primary human lung fibroblasts (phLFs) isolation was performed as previously described^47^. The phLFs were cultured in Dulbecco’s Modified Eagle’s medium/Nutrient mixture F12 medium (DMEM/F12) containing 20% (v/v) fetal calf serum (FCS), 100 mg/l streptomycin and 100 U/ml penicillin. Cells were synchronized before stimulation by culturing them for 24 hours in corresponding starvation medium supplemented with 0.1% (v/v) FCS and antibiotics. Cell stimulations were performed in fresh medium with identical composition as medium for cell synchronization. Cells were incubated at 37 °C, 5% CO_2_. For inhibitor studies, phLFs were seeded in 6 well-plates with a total of 2 × 10^5^ cells/well. 24 hours after seeding, cells were synchronized for 24 hours. Cells were pre-treated with different inhibitors for 1 hour (SB431542 – 10 μM; SC-514 – 50 μM; U0126 – 3 μM; SP600125 – 10 μM; 7-Z-Oxozeaneol – 500 nM) and subsequently treated with TGFβ1 (2 ng/ml) or TNFα (10 ng/ml) for 24 hours. For analysis of the time-dependent induction of WISP1, phLFs were seeded in 6 well-plates in a total of 2 × 10^5^ cells/well. 24 hours after seeding, cells were synchronized for 24 hours. Cells were subsequently treated with TGFβ1 (2 ng/ml) or TNFα (10 ng/ml) and treated for 8 to 48 hours. Supernatants were taken at 24 and 48 hours for WISP1 ELISA measurements. Cells were washed with cold PBS and thereafter taken for RNA isolation. To analyse the concentration-dependent induction of WISP1, phLFs were seeded in 6 well-plates with a total of 2 × 10^5^ cells/well. 24 hours after seeding, cells were synchronized for 24 hours. Cells were subsequently treated with TGFβ1 (0.5–10 ng/ml) or TNFα (10–100 ng/ml) and treated for 24 hours. Cells were washed with cold PBS and thereafter taken for RNA purification. Supernatants were stored at −80 °C until further use.

### WISP1 Luciferase promoter studies

The sequence of the WISP1 promoter region (2.5 kb from the transcription start site) was obtained from the USCS Genome Bioinformatics database (genome assembly: GRChg38; location: 133,188,539–133,191,039) and analysed using the Genomatix software version 3.4. The 2.5 kb element of the WISP1 promoter was cloned into the pGL4.10 vector. The phLFs were seeded in a 48 well plate at a density of 2.5 × 10^4^ cells/well in DMEM/F12 containing 20% (v/v) (FCS), 100 mg/l streptomycin and 100 U/ml penicillin. Cells were transfected 24 hours after seeding in serum-free Opti-MEM medium (Life Technologies, Darmstadt, Germany) plus Dulbecco’s Modified Eagle’s medium/Nutrient mixture F12 medium (DMEM/F12) containing 20% (v/v) fetal calf serum (FCS), 100 mg/l streptomycin and 100 U/ml penicillin (ratio 1:3) using 250 ng/ml of the vector construct including the 2.5 kb element of the WISP1 promoter region in combination with Lipofectamine LTX transfection reagent and PLUS reagent (Life Technologies, Darmstadt, Germany). Control transfections were performed using 250 ng of the pGL4.10 vector construct. The transfection mix was incubated in Opti-MEM medium for 30 minutes at room temperature. The transfection mix was then added to the wells on top of the refreshed starvation medium. Cells were transfected for 6 hours and thereafter cells were incubated in starvation medium overnight. Cells were subsequently stimulated for 24 hours with either Wnt3a (100 ng/ml), TGFβ1 (2 ng/ml) or TNFα (10 ng/ml) in DMEM/F12 medium supplemented with 0.1% FCS (v/v) and antibiotics. After 24 hours, cells were lysed and cell lysate suspension was used to determine the luciferase activity using the Berthold Tristar LB941 (luciferase reagent: Bright-Glo™ Luciferase Assay System, Promega, Mannheim, Germany). Measurements were performed in quadruplicates.

### WISP1 siRNA transfection

Primary human fibroblasts were seeded in different well plate formats (6 well: 2 × 10^5^ cells/well; 24 well: 5 × 10^4^ cells/well; 96 well: 5 × 10^3^ cells/well) and transiently transfected with a pool of specific double-stranded siRNAs targeted against the WISP1 transcript (On-Targetplus siRNA, J-010555-05, -07, -08; Dharmacon, Lafayette, Colorado, USA). Cells were transfected in serum-free Opti-MEM medium (Life Technologies, Darmstadt, Germany) plus DMEM/F12 containing 20% (v/v) FCS, 100 mg/l streptomycin and 100 U/ml penicillin (ratio 1:3) using 10 nM of siRNA in combination with Lipofectamine RNAiMax transfection reagent (Life Technologies, Darmstadt, Germany). Control transfections were performed using 10 nM ON-TARGETplus Non-targeting siRNAs (D-001810-10, Dharmacon, Lafayette, Colorado, USA). The siRNAs were incubated in Opti-MEM medium for 30 minutes at room temperature. The siRNA mix was then added to the wells and cell suspension was added on top. Cells were transfected overnight. Cells were subsequently stimulated for the indicated time-points with either TGFβ1 (2 ng/ml) or TNFα (10 ng/ml) in DMEM/F12 medium supplemented with 0.1% FCS and antibiotics. Supernatants were collected and cells were washed with cold PBS and thereafter taken for RNA purification. Supernatants were stored at −80 °C until further use.

### Treatment of phLFs with neutralizing αWISP1 antibody

Cells were seeded in 96 well format (5 × 10^3^ cells/well) in Dulbecco’s Modified Eagle’s medium/Nutrient mixture F12 medium (DMEM/F12) containing 20% (v/v) fetal calf serum (FCS), 100 mg/l streptomycin and 100 U/ml penicillin. After 24 hours cells were serum-starved (DMEM/F12 medium containing 0.1% FCS and antibiotics) for 24 hours. Prior to treatment, cells were pre-incubated with the neutralizing αWISP1 antibody (10µg/ml; R&D, AF1627) for 1 hour. Subsequently, cells were treated with or without 10 ng/ml IL-6 for 48 hours.

### Immunofluorescence staining

The phLFs were transfected as described above and cultured for 72 hours on poly-l-lysine-coated cover slips. Cells were fixed with acetone/methanol (1:1), permeabilized with 0.1% Triton X-100 in 1xPBS for 20 minutes and blocked with 5% (w/vol) bovine serum albumin (Sigma Aldrich) for 30 minutes. Cells were subsequently incubated with the respective primary antibody (PCNA, Zymed 18-0110, Vienna, Austria) at room temperature (RT) for 1 hour in PBS containing 0.1% (w/vol) BSA, followed by incubation with a fluorescently labeled secondary antibody (anti-mouse Alexa 488, Life Technologies). DAPI staining (Roche) was used to visualize cell nuclei.

### Immunoblotting

Cells were washed twice with phosphate-buffered saline (PBS; PAA Laboratories), lysed in T-PER lysis buffer (Thermo Fisher Scientific, Waltham, MA, US) supplemented with proteinase inhibitor cocktail tablets and PhosSTOP^TM^ (Roche), and lysates were centrifuged at 13 000 rpm at 4 °C. Supernatant was collected and protein concentration was determined using the Quick Start Bradford Dye Reagent according to the manufacturer’s instructions. 15 μg of total protein was separated on SDS-polyacrylamide gels and transferred to PVDF (Biorad, Hercules, CA, US). Membranes were blocked in 1x Roti®-Block (Roth, Karlsruhe, Germany) in TRIS-buffered saline containing 0.05% (v/v) Tween (TBST) (Applichem) and incubated with the primary antibody (Cyclin D1, 2978 P, New England Biolabs; Ipswich, MA, USA) at 4 °C overnight. The HRP-labeled secondary antibody (anti-rabbit-HRP antibody; GE Healthcare, Chalfont St Giles, UK) was applied after washing of the membrane in TBST. Proteins were visualized by autoradiography following incubation with SuperSignal West Dura Chemiluminescent Substrate (Thermo Fisher Scientific). β-actin served as loading control and was detected using a HRP-conjugated β-actin antibody (Sigma Aldrich).

### RNA isolation and reverse transcription real-time polymerase chain reaction

Total RNA was isolated from cells using the Peqlab Total RNA Kit (Peqlab, Erlangen, Germany) according to the manufacturer’s instructions. An amount of 1000 ng of RNA was used for cDNA synthesis as previously described^18^. The following primers were used: *Wnt1-inducible signaling protein 1* (forward: GGCATGAGGTGGTTCCTG; reverse: GGAGCTGGGGTAAAGTCCAT), *Interleukin 6* (forward: TTCCTGCAGAAAAAGGCAAAGA; reverse: CTGCGCAGAATGAGATGAGT), *Interleukin 8* (forward: CAGGAAGAAACCACCGGAAG; reverse: AACTGCACCTTCACACAGAG), *Serpine 1* (forward: GACATCCTGGAACTGCCCTA; reverse: GGTCATGTTGCCTTTCCAGT).

### WISP1 and Interleukin-6 enzyme-linked immunosorbent assay

Supernatants were taken from time-dependent TGFβ1 and TNFα WISP1-inductions or siRNA transfection assays and concentrated for WISP1 measurements by a factor of 5 using Amicon Ultra-0.5 centrifugal filter devices according to the manufacturer’s instructions (Merck Millipore, Amsterdam, The Netherlands) and the assay was performed according to the manufacturer’s instructions. Samples were then transferred to the WISP1 ELISA plate (DY1627; R&D, Minneapolis, Minnesota, USA). Samples for IL-6 measurements were diluted 1:10 in dilution buffer prior to transfer to the IL-6 ELISA plate (DY206; R&D, Minneapolis, Minnesota, USA) and the assay was performed according to the manufacturer’s instructions.

### WST1-Proliferation assay

Primary human lung fibroblasts were plated at a density of 5 × 10^3^ cells per well in a 96 wells plate. The next day cells were synchronized for 24 hours using DMEM/F12 medium supplemented with 0.1% FCS and antibiotics. Cells were stimulated with DMEM/F12 medium with 0.1% (v/v) FCS, DMEM/F12 medium with 10% (v/v) FCS or, or DMEM/F12 with 10% (v/v) FCS plus IL-6 (0.1–20 ng/ml) for 48 hours. Subsequently, 10 μl of WST-1 per 100 μl medium (10% v/v; Cat. No. 11 644 807 001, Roche Diagnostics GmbH, Mannheim, Germany) was added to each well and incubated for 2 hours. Plates were then measured using the Tecan Sunrise ELISA Reader at a wave length of 440 nm (reference wave length: 620 nm). Each condition was measured in triplicates.

### Cell counting

Primary human lung fibroblasts were plated at a density of 5 × 10^4^ cells per well in a 24 well plate. The next day cells were synchronized for 24 hours using DMEM/F12 medium supplemented with 0.1% FCS and antibiotics. Cells were stimulated with DMEM/F12 medium with 10% (v/v) for 48 hours. Subsequently, cells were washed with PBS, trypsinized and counted using a Neubauer chamber. Experiments were performed in duplicates.

### Correlation analysis

Data for the analysis were extracted from Lung Genomics Research Consortium (GSE47460 GPL4680) and correlated to diffusion capacity of the lung for carbon monoxide (DLCO) and the forced vital capacity (FVC) in human patients as a measure of disease severity. Only normal control patients and patients with confirmed IPF were used from the dataset.

### Statistical analysis

Data represent means ± SEM, from *n* independent experiments. Statistical significance of differences was evaluated by Student’s *t*-test or one-way ANOVA followed by a Newman-Keuls multiple comparison test, where appropriate. Correlation was evaluated by using the Pearson test. Differences were considered to be statistically significant when p < 0.05.

## Additional Information

**How to cite this article**: Klee, S. *et al.* WISP1 mediates IL-6-dependent proliferation in primary human lung fibroblasts. *Sci. Rep.* **6**, 20547; doi: 10.1038/srep20547 (2016).

## Supplementary Material

Supplementary Information
