# Supplementary material for: WISP1 mediates IL-6-dependent proliferation in primary human lung fibroblasts
Source: Sci Rep. 2016 Feb 12;6:20547. doi: 10.1038/srep20547 (PMC4751539; doi:10.1038/srep20547)
Supplement: Supplementary Information [file srep20547-s1.pdf]

WISP1 mediates IL-6-dependent proliferation in primary human lung fibroblasts

Klee S1, Lehmann M1, Wagner DE1, Baarsma HA1, Königshoff M1

1 – Comprehensive Pneumology Center, Helmholtz Zentrum München, Munich, Germany; Member of the German Center of Lung Research (DZL) and Ludwig-Maximilians-Universität, University Hospital Grosshadern

Corresponding author: melanie.koenigshoff@helmholtz-muenchen.de

Max-Lebsche Platz 31

81379 Munich

Germany

Tel: +49 89 3187 4668

Fax: +49 89 3187 4661

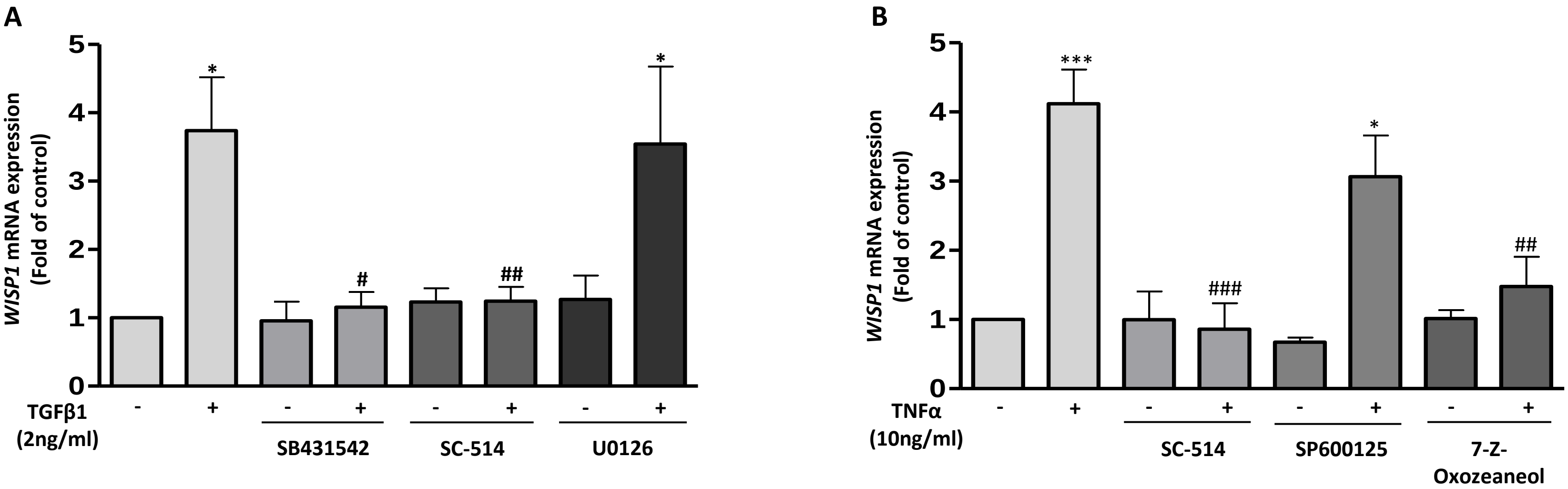

Supplementary Figure 1: TGFβ1- and TNFα induce *WISP1* in phLFs via NF-κB. The phLFs were treated for 24 hours with either (A) 2 ng/ml TGFβ1 in the absence or presence of the ALK5 inhibitor SB 431542 (10 µM), the IKKβ inhibitor SC-514 (10 µM) or the MEK1/2 inhibitor U126 (3 µM) or (B) with 10 ng/ml TNFα in the absence or presence of the IKKβ inhibitor SC-514 (10 µM), the JNK inhibitor SP 600125 (10 µM) or the TAK1 inhibitor 7-Z-Oxozeanol (500 nM). (n=3; *,# $p<0.05$; student´s t-test)

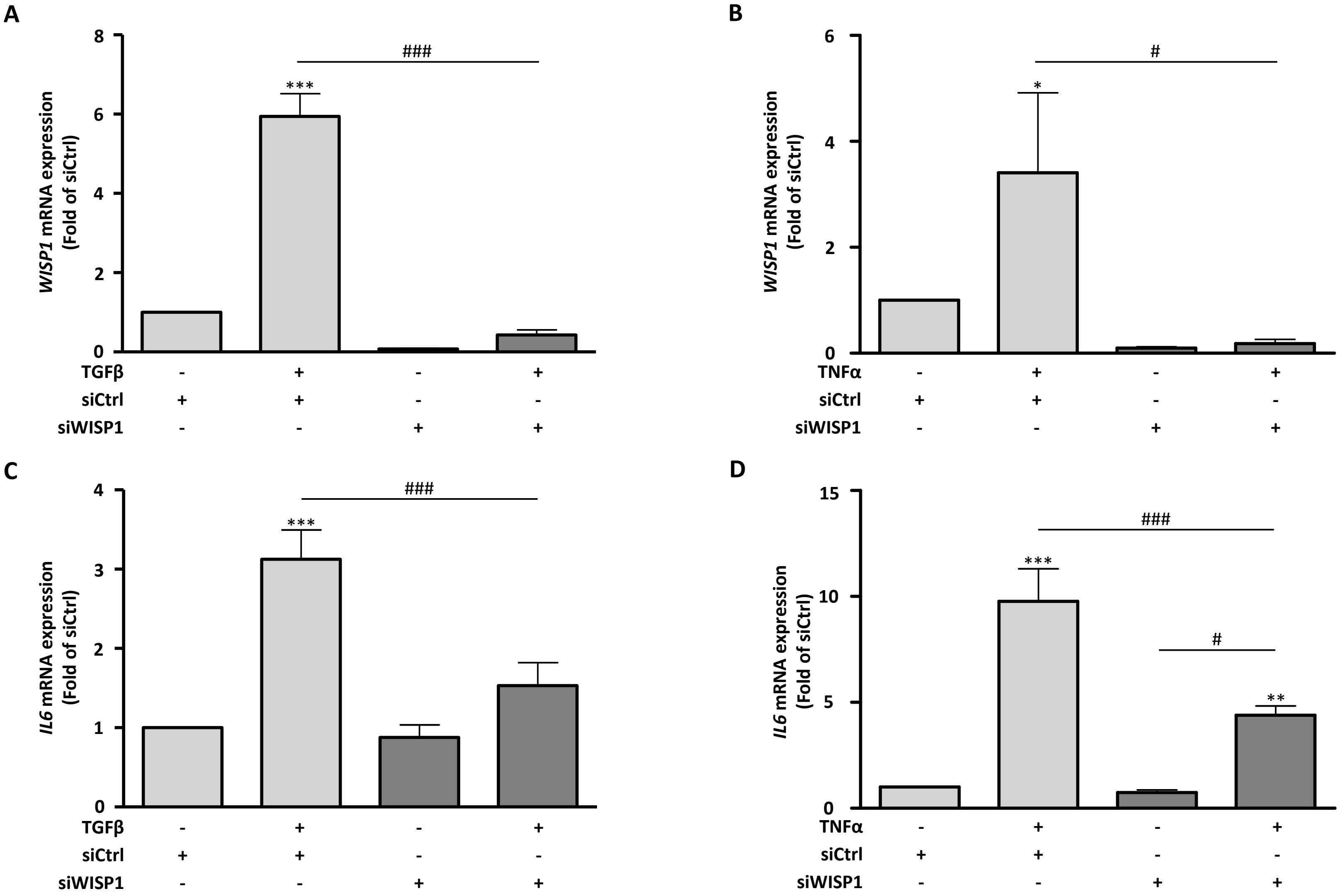

Supplementary Figure 2: WISP1 is required for *IL6* induction in phLFs derived from IPF patients. The phLFs were treated for 24 hours with either (A,C) 2 ng/ml TGFβ1 or (B,D) with 10 ng/ml TNFα in the absence or presence of the siRNA targeting WISP1 (siWISP1) or a non-targeting control siRNA (siCtrl). Effects on the expression of (A,C) *WISP1* and (B,D) *IL6* were analyzed using qPCR (n=3; *,# $p<0.05$; ** $p<0.01$; ***, ### $p<0.001$; 1-way Anova with Newman-Keuls Multiple comparison testing).

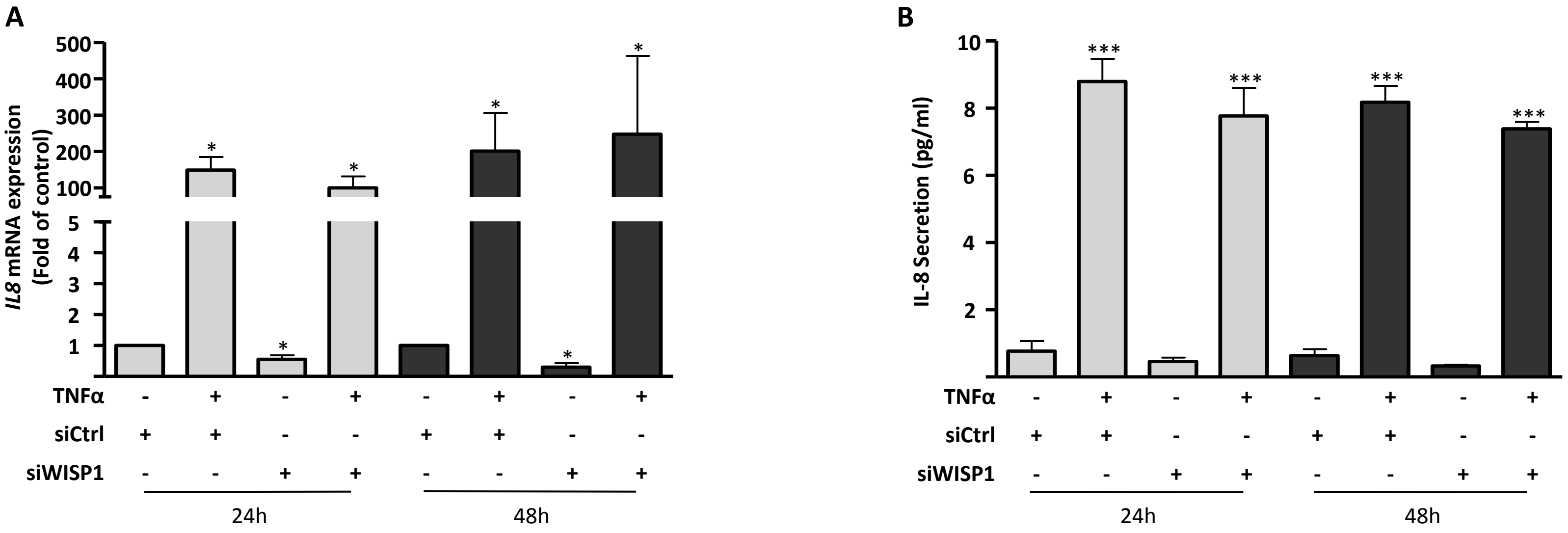

Supplementary Figure 3: Production of IL-8 upon TNFα stimulation is not affected by the absence of WISP1 in phLFs. The phLFs were treated for 24 and 48 hours with with 10 ng/ml TNFα in the absence or presence of the siRNA targeting WISP1 (siWISP1) or a non-targeting control siRNA (siCtrl). (A) The expression of *IL8* was measured by qPCR and (B) the secretion of IL-8 was analyzed using a multiplex ELISA (n=4; * p<0.05; *** p<0.001; 1-way Anova with Newman-Keuls Multiple comparison testing).

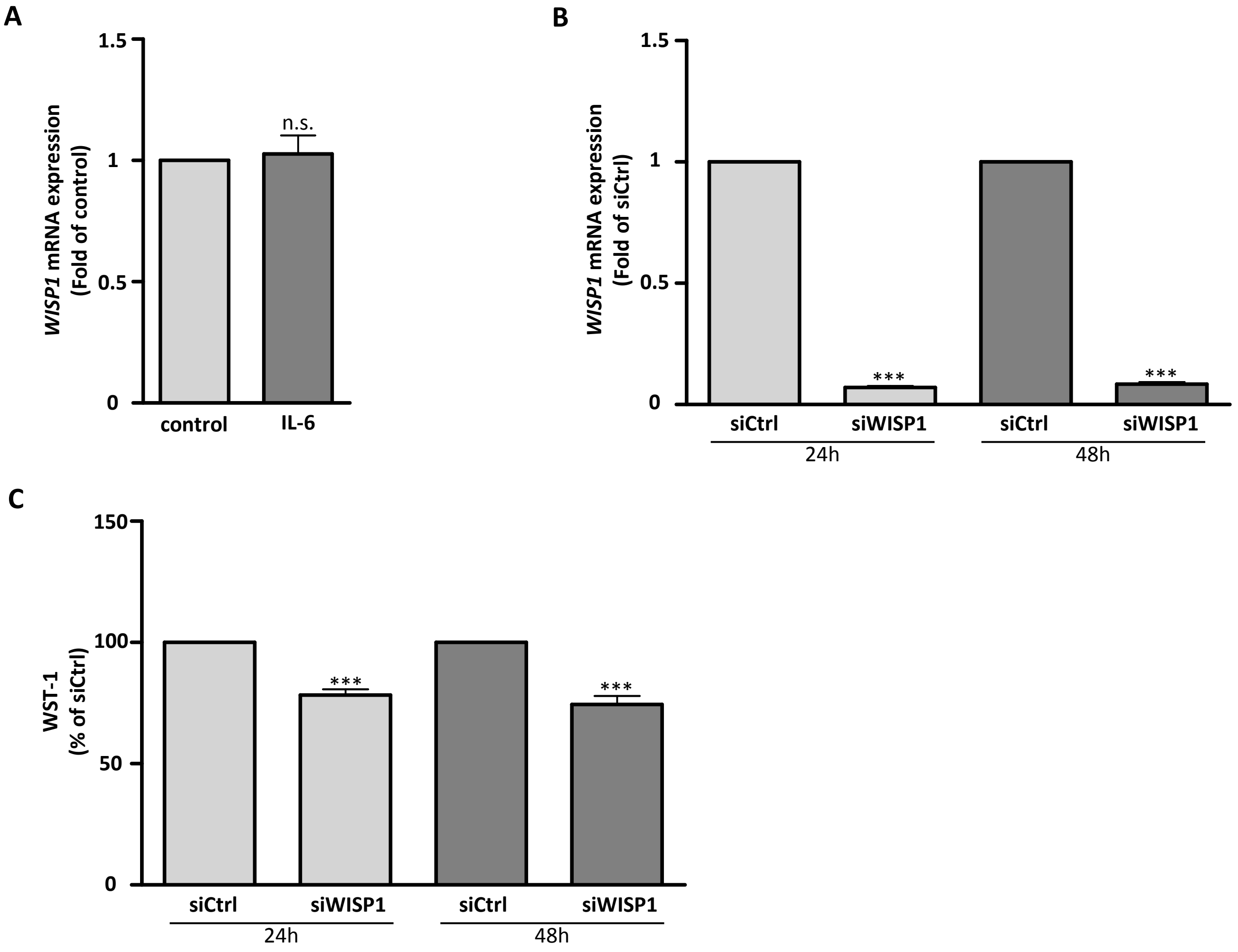

Supplementary Figure 4: *WISP1* is not induced by IL-6 stimulation in phLFs. (A) The phLFs were treated for 24 hours with 10 ng/ml IL-6 or (B,C) transfected with a WISP1-silencing siRNA (siWISP1) or a control siRNA (siRNA) and kept in 0.1% FCS-containing medium according to the stimulation protocol for 24 and 48h. (A,B) The expression of *WISP1* was measured by qPCR and (C) the metabolic activity of the phLFs was measured by WST-1 assay (n=7; *** $p<0.001$; 1-way Anova with Newman-Keuls Multiple comparison testing).

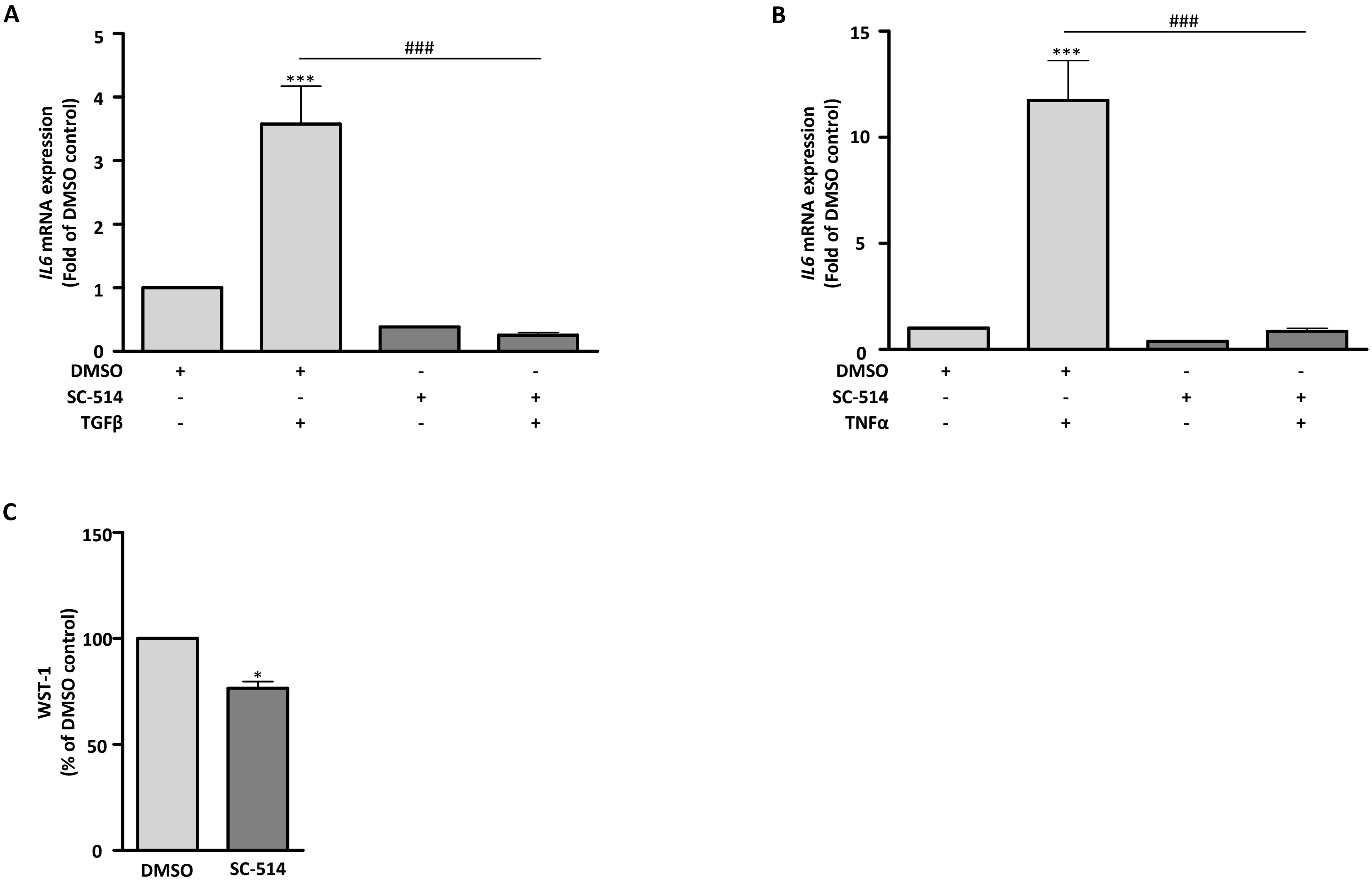

Supplementary Figure 5: The IKKβ inhibitor SC-514 abrogates the expression of *IL6* and reduces proliferation in phLF. The phLFs were treated for 24 hours with (A) 2 ng/ml TGFβ or (B) 10 ng/ml TNFα in the absence or presence of the IKKβ inhibitor SC-514 (50µM) and the expression of *IL6* was measured by qPCR. (C) Moreover, treatment of phLFs with SC-514 (50µM) for 48 hours and proliferation was assessed by WST-1 assay (n=3; ***,### $p<0.001$, 1-way Anova with Newman-Keuls Multiple comparison testing for (A) and (B); * $p<0.05$, students t-test for (C))

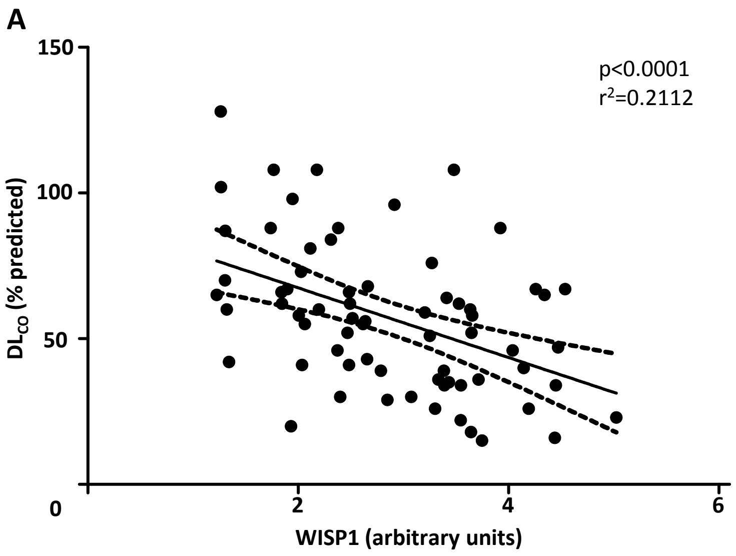

Supplementary Figure 6: WISP1 mRNA levels negatively correlate with the diffusion capacity of the lung for carbon monoxide (DL_{CO}) and the forced vital capacity (FVC) in human patients. The correlation of WISP1 and (A) the predicted DL_{CO} (N=68) or (B) the FVC (N=80) showed a significantly negative linear correlation. (dashed line = 95% CI; Data extracted from the LGRC GSE47460 GPL4680)

TGFβ treatment

	siCtrl (pg/ml)	siWISP1 (pg/ml)	siCtrl + TGFβ (pg/ml)	siWISP1 + TGFβ (pg/ml)
IL-6	0.76 ± 0.10	0.24 ± 0.05*	2.33 ± 0.10*	0.9 ± 0.17#
IL-8	0.75 ± 0.42	0.39 ± 0.09	0.53 ± 0.25	0.33 ± 0.09
MCP1	1.57 ± 0.44	0.83 ± 0.31	0.5 ± 0.14*	0.31 ± 0.08*
IFNγ	0.07 ± 0.03	0.01 ± 0.01*	0.11 ± 0.02	0.02 ± 0.01*
GM-CSF	n.d.	n.d.	n.d.	n.d.
IL-7	0.01 ± 0.00	0.01 ± 0.01	0.01 ± 0.00	0.01 ± 0.01
TNFα	0.01 ± 0.00	n.d.	0.01 ± 0.00	n.d.
GCSF	0.01 ± 0.00	n.d.	0.01 ± 0.00	n.d.
IL-4	0.01 ± 0.00	n.d.	0.01 ± 0.00	n.d.
MIP1b	n.d.	n.d.	n.d.	n.d.
IL-2	n.d.	n.d.	n.d.	n.d.
IL-17	n.d.	n.d.	n.d.	n.d.
IL-10	n.d.	n.d.	n.d.	n.d.
IL-12	n.d.	n.d.	n.d.	n.d.
IL-13	n.d.	n.d.	n.d.	n.d.
IL-5	n.d.	n.d.	n.d.	n.d.
IL-1b	n.d.	n.d.	n.d.	n.d.

Supplementary Table 1: The secretion of different cytokines by phLFs was measured after TGFβ1 (2 ng/ml) stimulation *in vitro* for 24 hours using a multiplex ELISA kit (Bio-Plex Pro™ Human Cytokine 17-plex). (n=4; *,# $p<0.05$; 1-way ANOVA followed by Neuman-Keuls multiple comparison test)

TNFα treatment

	siCtrl (pg/ml)	siWISP1 (pg/ml)	siCtrl + TNFα (pg/ml)	siWISP1 + TNFα (pg/ml)
IL-6	0.77 ± 0.16	0.26 ± 0.05*	13.86 ± 3.97*	5.61 ± 0.92*,#
IL-8	0.77 ± 0.52	0.47 ± 0.19	8.79 ± 1.17*	7.77 ± 1.45*
MCP1	1.52 ± 0.41	0.90 ± 0.38	2.85 ± 0.60*	2.35 ± 0.23*
IFNγ	0.08 ± 0.03	0.03 ± 0.02*	0.39 ± 0.02*	0.28 ± 0.02*,#
GM-CSF	n.d.	n.d.	0.24 ± 0.08*	0.06 ± 0.07#
IL-7	0.01 ± 0.00	n.d.	0.01 ± 0.01	0.01 ± 0.00
TNFα	0.01 ± 0.00	n.d.	0.04 ± 0.00	0.03 ± 0.00
GCSF	0.01 ± 0.00	0.05 ± 0.01	n.d.	0.05 ± 0.01
IL-4	0.01 ± 0.00	n.d.	0.02 ± 0.01	0.02 ± 0.00
MIP1b	n.d.	n.d.	0.05 ± 0.01	0.04 ± 0.01
IL-2	n.d.	n.d.	0.04 ± 0.00	0.02 ± 0.00
IL-17	n.d.	n.d.	0.05 ± 0.01	0.03 ± 0.02
IL-10	n.d.	n.d.	n.d.	n.d.
IL-12	n.d.	n.d.	n.d.	n.d.
IL-13	n.d.	n.d.	n.d.	n.d.
IL-5	n.d.	n.d.	n.d.	n.d.
IL-1b	n.d.	n.d.	n.d.	n.d.

Supplementary Table 2: The phLFs were treated with 10ng/ml TNFα for 24 hours in vitro and different cytokines were measured using a multiplex ELISA kit (Bio-Plex Pro™ Human Cytokine 17-plex) (n=4; *,# $p<0.05$; 1-way ANOVA followed by Neuman-Keuls multiple comparison test)
